# Acupuncture as prophylaxis for menstrual-related migraine: study protocol for a multicenter randomized controlled trial

**DOI:** 10.1186/1745-6215-14-374

**Published:** 2013-11-06

**Authors:** Xiao-Zhe Zhang, Lei Zhang, Jia Guo, Lin Zeng, Yi Yang, Tao Zhang, Guang-Xia Shi, Hui-Lin Liu, Lin-Peng Wang

**Affiliations:** 1 Department of Pain Management, Beijing Tiantan Hospital Affiliated to Capital Medical University, Beijing, China; 2 Acupuncture and Moxibustion Department, Beijing Hospital of Traditional Chinese Medicine affiliated to Capital Medical University, 23 Meishuguanhou Street, Dongcheng District, Beijing 100010, China; 3 Department of Traditional Chinese Medicine, Peking University Third Hospital, Beijing, China; 4 Research Center of Clinical Epidemiology Affiliated to Peking University, Beijing, China

## Abstract

**Background:**

Menstrual-related migraine is a common form of migraine affecting >50% of female migraineurs. Acupuncture may be a choice for menstrual-related migraine, when pharmacological prophylaxis is not suitable. However, the efficacy of acupuncture has not been confirmed. We design and perform a randomized controlled clinical trial to evaluate the efficacy of acupuncture compared with naproxen in menstrual-related migraine patients.

**Methods/Design:**

This is a multicenter, single blind, randomized controlled clinical trial. A total of 184 participants will be randomly assigned to two different groups. Participants will receive verum acupuncture and placebo medicine in the treatment group, while participants in the control group will be treated with sham acupuncture and medicine (Naproxen Sustained Release Tablets). All treatments will be given for 3 months (menstrual cycles).

The primary outcome measures are the change of migraine days inside the menstrual cycle and the proportion of responders (defined as the proportion of patients with at least a 50% reduction in the number of menstrual migraine days). The secondary outcome measures are the change of migraine days outside the menstrual cycle, duration of migraine attack, the Visual Analogue Scale (VAS), and intake of acute medication. The assessment will be made at baseline (before treatment), 3 months (menstrual cycles), and 4 months (menstrual cycles) after the first acupuncture session.

**Discussion:**

The results of this trial will be helpful to supply the efficacy of acupuncture for menstrual-related migraine prophylaxis.

**Trial registration:**

ISRCTN: ISRCTN57133712

## Background

Migraine is a common neurological disorder. Population-based studies suggest that 6% to 7% of men and 15% to 18% of women experience migraine [1,2]. More than 50% of female patients report that their migraines are associated with the menses [3]. Attack onset is usually before the age of 20 years, with peak prevalence between the ages of 25 and 55 years, declining with menopause [4-6]. Increasing evidence links menstrual migraine to the female sex hormones [3,7].

Approximately 14% of female migraineurs have migraine only during menstruation (pure menstrual migraine (PMM)) while 60% suffer from migraine at both menses and other times during the menstrual cycle (menstrual-related migraine (MRM)) [8]. Most of the data in the literature report that MRM causes significant limitations of daily activities (for example, nausea, vomiting, and photo-phonophobia), and the attacks are generally longer, more severe, and less drug-responsive than non-menstrual ones [9,10].

Given the particular clinical picture that characterizes MRM, it is not surprising that the attacks are difficult to treat. The options available for the treatment of MRM include acute therapy and prophylaxis (short-term preventive therapy and long-term preventive therapy) [11-14]. Acute therapy is initiated first. As MRM is unique in its predictability, treatment can be targeted to the period of time when patients are most likely to experience migraine. The treatment window for menstrual migraine occurs between 2 days before and 3 days after the onset of menses [12]. The goals of prophylactic strategies are to reduce attack frequency, severity and duration, improve responsiveness of treatment for acute attacks, improve function, and reduce disability [13]. Prophylactic medications include non-steroidal anti-inflammatory drugs (NSAIDs), triptans, estrogen, magnesium, dihydroergotamine (DHE), methysergide, and vitamin E [14]. Caution should be taken when the drug is used within the same month for treating migraine attacks occurring during menses and outside the menstrual period due to the risk of medication-overuse headache, a risk which is, however, common to all medications used for menstrual-migraine prophylaxis or treatment [14].

Acupuncture, which is one of the main treatment modalities of Traditional Chinese Medicine (TCM), has been used for both the prevention and treatment of diseases for over 2,000 years. Several studies [15-18] have already reported the encouraging results in the therapy for migraine by acupuncture. A German randomized controlled trial (*n* = 794) showed that 11 acupuncture treatments given within 6 weeks were at least as effective as a β-blocker taken daily over 6 months [15]. Yang et al. found that acupuncture treatment was more effective and safer than topirmarate [16]. Wang and co-authors reported that acupuncture was more effective than flunarizine in decreasing the duration of migraine attacks [17]. Therefore, acupuncture should be considered as an option for patients with migraine [18]. Although the physiological mechanism is still unclear [19], in the last 40 years, many theoretically plausible models have been developed, such as the principle of counter-irritation diffuse noxious inhibitory control (DNIC) and the endorphin hypothesis Melzack’s gate control theory [20-22]. DNIC is regarded as a possible factor explaining central pain modulation through acupuncture [23,24]. The endorphin hypothesis serves to explain a short-term analgesic effect of acupuncture, which is considered very robust.

However, based on a review of published literatures, the guidelines did not make treatment recommendation for acupuncture, because this therapy lacked adequate randomized controlled trials to support its effect [25].

When reviewing the evidence of acupuncture for MRM, only one published randomized controlled trial could be found [26]. The objectives of that study were to introduce a new method for controlled trials of acupuncture for headache and to examine the role of needling *per se*. No significant differences were found between the verum group and the placebo group.

In this study, we will perform a randomized controlled trial to investigate the efficacy of acupuncture treatment as prophylaxis for MRM.

## Methods/Design

### Ethics

The trial protocol is in accordance with the principles of the Declaration of Helsinki and has been approved by the Research Ethical Committee of Beijing Hospital of Traditional Chinese Medicine Affiliated to Capital Medical University on 10 May 2012 (ref: 201212). This trial was registered at Current Controlled Trials (ISRCTN57133712). Each participant will be notified regarding the study protocol. Written informed consent will be obtained from each participant.

### Participants

#### Population

A target sample of 184 participants will be recruited in acupuncture clinic from the following four hospitals: Beijing Traditional Chinese Medicine Hospital Affiliated to Capital Medical University, Peking University Third Hospital, Beijing Tiantan Hospital Affiliated to Capital Medical University, and Xiyuan Hospital affiliated with China Academy of the Chinese Medical Sciences. The trial will be exercised from December 2011 to December 2013.

#### Sample size

According to the previous pilot study [27], the number of migraine days after treatment in acupuncture group is 3.1 ± 2.7 days, and the control group is 5.2 ± 4.4 days.

Based on 0.8 power to detect a significant difference (α = 0.01, two-sided), 73 participants will be required for each group, which is calculated by PASS 2008. Allowing for a 20% withdrawal rate, we will plan to enroll a total of 184 participants with 92 participants in each group.

#### Recruitment of participants

Two strategies will be used to recruit participants with MRM. One is to recruit participants in outpatient clinics from the four hospitals mentioned above. The other is to show recruitment posters outside the clinics. The posters will contain brief introductions about the population needed to be involved, the free acupuncture treatments offered to eligible participants, and the contact information of the researcher.

#### Baseline assessment

A baseline registration will be undertaken before treatment. At baseline, participants will be required to provide a review of migraine history covering at least the preceding 3 months (menstrual cycles), medical history in general, and the latest medication history.

#### Inclusion criteria

Participants who meet all the following requirements will be allowed for enrollment:

1. Diagnosis of MRM [28]: MRM without aura (code A1.1.2), in which migraine without aura always occurs on days 1 ± 2 of menstruation in at least two out of three menstrual cycles, and at other times due to different triggering factors or for no apparent specific reason (International Classification of Headache Disorders - second edition [ICHD-II])

2. Regular menstrual cycle

3. Predictable onset of inside-cycle migraine

4. Repeated migraine attacks, frequency of non-menstrual migraine is more than once a month

5. Written informed consent

#### Exclusion criteria

1. Chronic migraine, tension-type headache, cluster headache, and other primary headaches

2. Secondary headache and other neurological diseases

3. Relatively severe systemic diseases (cardiovascular disease, acute infectious disease, hematopathy, endocrinopathy, allergy, and methysis)

4. Headache caused by otorhinolaryngology diseases or intracranial pathological changes

5. Oral contraceptives, pregnancy, or lactation period

6. Use of prophylactic migraine medication in the last 3 months

7. Participation in another clinical trial

### Randomization and blinding

This is a multicenter (four hospitals in China), randomized controlled clinical trial. The central randomization will be performed by the Research Center of Clinical Epidemiology affiliated to Peking University in China, which use block randomization to generate the random allocation sequence and prepare predetermined computer-made randomization opaque sealed envelopes. The envelopes will be numbered consecutively and connected into a strain. Each envelope will be separated from the strain and then opened in sequence only after baseline period when the participants are registered in the trial.

In this study, the participants will be informed that they have a 50% chance of being allocated in either of the two groups: verum acupuncture plus placebo medication group and sham acupuncture plus true medication group. The placebo medication will be identical to true medication and sham acupuncture will produce same stimulation as verum acupuncture; hence participants will be blinded to the group allocation. A credibility of blinding test will be carried out in the middle of treatment phase and at the end of treatment phase. Furthermore, outcome assessors and personnel who will deal with data collection and data analysis will be blinded throughout the entire trial. The acupuncturist cannot be blinded due to the nature of the intervention, but they will be trained not to communicate with the participants or outcome assessors regarding treatment procedures and responses.

### Procedure

According to the predetermined randomization envelopes, participants will be randomly allocated to acupuncture group or control group (see Additional file 1: Figure S1). Participants will be asked to record headaches and usage of acute medication in a headache diary from the baseline period to the fourth month (menstrual cycle) after randomization. The headache diary will be collected separately from four hospitals in the fourth month (menstrual cycle) after randomization by blinded telephone interviewers (see Additional file 2: Table S1).

### Interventions

Interventions are selected according to records in ancient and modern books and results of previous research about treating migraine with acupuncture [17,27]. All participants will go through a standardized interview and receive more information about the study. Acupuncturists for both groups have >20 years of clinical experience and an acupuncture license from the Chinese medicine practitioner license from the Ministry of Health of the People’s Republic of China. Before performing this trial, all acupuncturists will receive special training about the purpose and content of the trial, treatment strategies, and quality control. The treatments are fully documented in accordance with the STRICTA [29] and good clinical practice guidelines (GCP).

All participants will receive treatment for 3 months (menstrual cycles). In the treatment group, participants will receive two acupuncture sessions per week (including during menstruation) for preventive treatment on migraine, and at least three additional acupuncture sessions for premenstrual conditioning during 10 days before menstruation, while placebo medicine will be given. In the control group, participants will be given medicine (Naproxen Sustained Release Tablets) and two sham acupuncture sessions per week.

Each session will last for 30 min. The number of needles will be from 10 to 12 in each session for both groups. Sterile needles for single use (Hwato Needles, made in Suzhou, China) are used in this trial. 30-gauge (0.3 mm in diameter), 40 mm-long needles are used for limb and abdomen points and 32-gauge (0.25 mm in diameter), 25 mm-long needles for head points. All needles will be inserted 10 to 15 mm in depth and manually manipulated by rotation methods to produce a characteristic sensation known as De Qi (feeling of needle sensation refers to tenseness around the needle felt by the practitioner and numbness, distension, soreness, and heaviness around the point felt by the patient).

The placebo medicine will have exactly the same appearance as true medicines (Naproxen Sustained Release Tablets). Both Naproxen Sustained Release Tablets and placebo will be taken 0.5 g once per day, from the third day (± 1 day accepted) before menstruation comes to an end. All participants will be permitted to treat acute headaches as required, but the type and dosage of used medications should be recorded in the headache diary.

#### The points used in the treatment group

1.1 Preventive Treatment on Migraine by Acupuncture

Main points: DU20 (Baihui), DU24 (Shenting), GB13 (Benshen), GB8 (Shuaigu), SJ20 (Jiaosun), and GB20 (Fengchi)

Additional points can be chosen according to syndrome differentiation of meridians: Shaoyang headache (TE-GB): SJ5 (Waiguan), GB34 (Yanglingquan); Yangming headache (LI-ST): LI4 (Hegu), ST44 (Neiting); Taiyang headache (SI-BL): BL60 (Kunlun), SI3 (Houxi); Jueyin headache (PC-LR): LR3 (Taichong), GB40 (Qiuxu); Nausea and vomiting: PC6 (Neiguan); Dysphoria and susceptibility to rage: LR3 (Taichong)

1.2 Premenstrual Conditioning by Acupuncture

Points: KI12 (Dahe), RN3 (Zhongji), ST29 (Guilai)

#### The points used in the control group

To make the quantity of stimulus uniform between two groups, the decision is made to use the same kind, size, and number of needles for control group as for treatment group. The procedure for developing the sham acupuncture protocol contains three steps.

1. Literature search: Based on the search and analyses of 26 ancient Chinese books of acupuncture, three Chinese acupuncture textbooks and >100 acupuncture research literatures, the points with no reference to therapeutic effects on headache or menstruation have been enumerated.

2. Point selection: To avoid effects on headache and effects of alleviating pain, the points in the head, trunk, hands, and feet are excluded. And then 15 points (see Additional file 3: Table S2) without effects on headache or menstruation are extracted and these points are defined as sham points, which will be used in the control group.

3. Sham points subgroup: 15 sham points will be randomly assigned to three subgroups of control groups which are labeled from B to D and will be recorded in the predetermined computer-made randomization sealed envelope. Each subgroup has two points on arms and three points on legs (see Additional file 4: Table S3). A participant who belongs to the control group will be assigned to one of subgroups and the five points in this subgroup will be used on this participant in the whole treatment period.

### Outcome measures

The efficacy of acupuncture for migraine prophylaxis will be assessed by the following primary outcome measures:

1. The change of migraine days inside the menstrual cycle [15]

2. The proportion of responders (defined as the proportion of patients with at least a 50% reduction of the number of menstrual migraine days) [15]

The secondary outcome measures include:

1. The change of migraine days outside the menstrual cycle [15]

2. Duration of migraine attack [15]

3. Visual analogue scale (VAS) for pain, ranging from 0 (no pain) to 10 (worst pain ever) [30]

4. Intake of acute medication [15]

The outcome measures above will be assessed at baseline (before treatment), 3 months (menstrual cycles) after the first acupuncture session, and four months (menstrual cycles) after the first acupuncture session.

The participants will be asked to fill in headache diaries from baseline period to the fourth month (menstrual cycle) after randomization. In the diaries, the participants will record on diary cards details of their migraine attacks, including the time of headache attack and cease, intensity, frequency, location, and cause of the headache and its associated symptoms in each migraine attack. The participants will document whether or not they take the acute medicine. The name, dosage of the medicine, time of taking medicine, time of pain relief, and side effects of the medicine will also be recorded by the participants if they take the medicine. The participants will also complete calendar cards to indicate the days on which the menstruation occurs and which they receive acupuncture treatment.

The participants will report the adverse events they experience, including discomfort or bruising at the sites of needle insertion, nausea, or feeling faint after each treatment.

The reason for drop-out or withdrawal, as well as the participant’s compliance, will be documented in CRF during the 3 months of treatment and 1 month of follow-up.

### Statistical analysis

The analyses will be performed on an intention-to-treat basis, with missing data replaced by last measure values. The *P* value for statistical significance is defined as *P* <0.05 in all comparisons, and all statistical testing is two-sided. Analysis of variance (ANOVA) for repeated measures is used to compare the two groups. Comparisons within groups for the outcomes in each one of the time point are done using the Tukey’s post hoc test of Multivariate Analysis of Variance (MANOVA). As for proportions, a chi-square test is applied. Stratified analysis within four different centers will be performed to control confounding factor if necessary.

Data analysis will be conducted by statisticians who are independent from the research team. Every analysis is conducted by using the SPSS software version 13.0 (SPSS, Chicago, IL, USA).

## Discussion

We have presented the design and the protocol for a randomized controlled trial of acupuncture compared with naproxen in the prophylactic therapy for patients with MRM. Completion of this trial will help to supply the evidence on the efficacy of acupuncture for MRM prophylaxis.

During the preparation period of this trial, we spent plenty of time designing the control group, since appropriate control is crucial for a high-quality randomized controlled trial. One major issue is whether sham acupuncture should be used. As the primary consideration of this trial is to clarify whether acupuncture is effective for MRM prophylaxis, we all agree that a pragmatic design is more suitable, which requires standard care rather than placebo controlled trials [31]. However, in China, because of the common concept of drug dependence, a large proportion of patients are unwilling to use medication, despite a confirmed diagnosis of migraine. Acupuncture has been commonly used in clinical practice in China. Patients usually expect to receive acupuncture treatment when they participate in the trial. To improve compliance of participants, sham acupuncture will be adopted in the control group while placebo medicine will be used in acupuncture group.

Naproxen is a drug classified as NSAIDs, which is considered safe and effective for the prophylactic treatment of menstrual migraine [32-34]. Therefore, we compare the efficacy of acupuncture with naproxen, which is chosen as a standard drug therapy.

Clinical acupuncture research is complicated for several reasons, of which the placebo issue is the most intriguing one [35,36]. Therefore, the American Headache Society (AHS) has stressed the need for developing new methods for controlled studies of acupuncture in treating headache [37].

The choice of sham points developed in our trial is based on international considerations about the methodological problems of sham acupuncture [38]. To avoid any influence on MRM, we searched points without effect on headache or menstruation from both ancient and modern literatures. This method of selecting sham points can be seen in Alecrim [39], nevertheless, our searching scope is more extensive. We have searched many Chinese articles, particularly ancient books, which have not yet been translated into English. Last but not least, there are 15 points without effects on headache or menstruation extracted, but only five points will be applied to each patient in control group. Points, chosen and made up as a control subgroup, are determined by computer-made randomization. This process of confirming sham points could further eliminate potential effects on MRM.

However, this study has several methodological limitations. Previous randomized controlled trials suggest that there is no obvious difference between verum and sham acupuncture groups, even though they are effective compared with no treatment [40,41]. Repetitive relaxation and being cared for may be the reasons. In the present study, these factors have been standardized as strictly as possible and the selection of sham points is different from previously published trials. We still cannot rule out the physiological effects of our control intervention design. Another limitation is that the therapist is not blinded in this trial. It is almost impossible to have genuine double blinding in acupuncture trials, because acupuncturists have to control participant’s perception of needling. This study does not allow us to determine whether the observed effectiveness is caused by placebo effects, intensity of provider contact, or a physiologic effect of needling. Therefore, a bias due to unblinding cannot be ruled out. In addition, the sample size in the present study is relatively small.

## Trial status

The trial is currently in the recruitment phase.

## Competing interests

The authors declare that they have no competing interests.

## Authors’ contributions

XZZ conceived this study and prepared the initial protocol. LZ drafted the manuscript and participated in design of the study. JG and LZ participated in rewriting the manuscript. YY, GXS, and TZ participated in the development of the acupuncture point protocol. HLL and LPW made amendments and participated in design of the trial protocol. All authors read and approved the final manuscript.

## Supplementary Material

Additional file 1: Figure S1 Study flow chart. Click here for file

Additional file 2: Table S1 Time to visit and data collection. Click here for file

Additional file 3: Table S2 Fifteen points without effects on headache or menstruation. Click here for file

Additional file 4: Table S3 The sham points in the control group. Click here for file

## References

[B1] Stewart WF Shechter A Rasmussen BK Migraine prevalence. A review of population-based studies Neurology 1994 Suppl 4 S17 S23 8008222

[B2] Lipton RB Stewart WF Diamond S Diamond ML Reed M Prevalence and burden of migraine in the United States: data from the American migraine study II Headache 2001 41 646 657 10.1046/j.1526-4610.2001.041007646.x 11554952

[B3] Granella F Sances G Allais G Nappi RE Tirelli A Benedetto C Brundu B Facchinetti F Nappi G Characteristics of menstrual and nonmenstrual attacks in women with menstrually related migraine referred to headache centres Cephalalgia 2004 24 707 716 10.1111/j.1468-2982.2004.00741.x 15315526

[B4] Stewart WF Lipton RB Celentano DD Reed ML Prevalence of migraine headache in the United States. Relation to age, income, race, and other sociodemographic factors JAMA 1992 267 64 69 10.1001/jama.1992.03480010072027 1727198

[B5] Pryse-Phillips W Findlay H Tugwell P Edmeads J Murray TJ Nelson RF A Canadian population survey on the clinical, epidemiologic and societal impact of migraine and tension-type headache Can J Neurol Sci 1992 19 333 339 1393842

[B6] Epstein MT Hockaday JM Hockaday TD Migraine and reporoductive hormones throughout the menstrual cycle Lancet 1975 1 543 548 47017 10.1016/s0140-6736(75)91558-5

[B7] Newman LC Lipton RB Lay CL Solomon S A pilot study of oral sumatriptan as intermittent prophylaxis of menstruation-related migraine Neurology 1998 51 307 309 10.1212/WNL.51.1.307 9674831

[B8] Nattero G Menstrual headache Adv Neurol 1982 33 215 226 7198864

[B9] Couturier EG Bomhof MA Neven AK van Duijn NP Menstrual migraine in a representative Dutch population sample: prevalence, disability and treatment Cephalalgia 2003 23 302 308 10.1046/j.1468-2982.2003.00516.x 12716349

[B10] MacGregor EA Hackshaw A Prevalence of migraine on each day of the natural menstrual cycle Neurology 2004 63 351 353 10.1212/01.WNL.0000133134.68143.2E 15277635

[B11] Lay CL Payne R Recognition and treatment of menstrual migraine Neurologist 2007 13 197 204 10.1097/NRL.0b013e31805c746f 17622911

[B12] Silberstein SD Hutchinson SL Diagnosis and treatment of the menstrual migraine patient Headache 2008 Suppl 3 S115 S123 19076657 10.1111/j.1526-4610.2008.01309.x

[B13] Evidence-based guidelines for migraine headache in the primary care setting: pharmacological management for prevention of migrainehttp://www.doc88.com/p-909539015858.html

[B14] MacGregor EA Prevention and treatment of menstrual migraine Drugs 2010 70 1799 1818 10.2165/11538090-000000000-00000 20836574

[B15] Diener HC Kronfeld K Boewing G Lungenhausen M Maier C Molsberger A Tegenthoff M Trampisch HJ Zenz M Meinert R Efficacy of acupuncture for the prophylaxis of migraine: a multicentre randomised controlled clinical trial Lancet Neurol 2006 5 310 316 10.1016/S1474-4422(06)70382-9 16545747

[B16] Yang CP Chang MH Liu PE Li TC Hsieh CL Hwang KL Chang HH Acupuncture versus topiramate in chronic migraine prophylaxis: a randomized clinical trial Cephalalgia 2011 31 1510 1521 10.1177/0333102411420585 22019576

[B17] Wang LP Zhang XZ Guo J Liu HL Zhang Y Liu CZ Yi JH Wang LP Zhao JP Li SS Efficacy of acupuncture for migraine prophylaxis: a single-blinded, double-dummy, randomized controlled trial Pain 2011 152 1864 1871 10.1016/j.pain.2011.04.006 21616596

[B18] Linde K Allais G Brinkhaus B Manheimer E Vickers A White AR Acupuncture for migraine prophylaxis Cochrane Database Syst Rev 2009 1 D1218 10.1002/14651858.CD007587 PMC3099266 19160338

[B19] Endres HG Diener HC Molsberger A Role of acupuncture in the treatment of migraine Expert Rev Neurother 2007 7 1121 1134 10.1586/14737175.7.9.1121 17868011

[B20] Melzack R Wall PD Pain mechanisms: a new theory Science 1965 150 971 979 10.1126/science.150.3699.971 5320816

[B21] Carlsson C Acupuncture mechanisms for clinically relevant long-term effects–reconsideration and a hypothesis Acupunct Med 2002 20 82 99 10.1136/aim.20.2-3.82 12216606

[B22] Pielsticker A Haag G Zaudig M Lautenbacher S Impairment of pain inhibition in chronic tension-type headache Pain 2005 118 215 223 10.1016/j.pain.2005.08.019 16202520

[B23] Rossi P Serrao M Perrotta A Pierelli F Sandrini G Nappi G Neurophysiological approach to central pain modulation in primary headaches J Headache Pain 2005 6 191 194 10.1007/s10194-005-0182-1 16362661 PMC3452008

[B24] Irnich D Beyer A Neurobiological mechanisms of acupuncture analgesia Schmerz 2002 16 93 102 10.1007/s004820100094 11956894

[B25] Campbell JK Penzien DB Wall EM Evidence-based guidelines for migraine headache: behavioral and physical treatmentshttp://www.neurology.org Accessed April 25, 2000

[B26] Linde M Fjell A Carlsson J Dahlof C Role of the needling per se in acupuncture as prophylaxis for menstrually related migraine: a randomized placebo-controlled study Cephalalgia 2005 25 41 47 10.1111/j.1468-2982.2004.00803.x 15606569

[B27] Li C Liu H Yang C Clinical observation of acupuncture as prophylaxis for menstrually related migraine (MRM) Beijing Journal of Traditional Chinese Medicine 2011 30 617 618

[B28] Headache Classification Subcommittee of the International Headache Society The International Classification of Headache Disorders: 2nd edition Cephalalgia 2004 Suppl 1 9 160 10.1111/j.1468-2982.2003.00824.x 14979299

[B29] MacPherson H White A Cummings M Jobst KA Rose K Niemtzow RC Standards for reporting interventions in controlled trials of acupuncture: the STRICTA recommendations J Altern Complement Med 2002 8 85 89 10.1089/107555302753507212 11890439

[B30] Zhao Y The measurements and assessments of pain (Chinese) Chinese Journal of Clinical Rehabilitation 2002 6 2347 2352

[B31] Linde K Vickers A Hondras M ter Riet G Thormahlen J Berman B Melchart D Systematic reviews of complementary therapies - an annotated bibliography. Part 1: acupuncture BMC Complement Altern Med 2001 1 3 10.1186/1472-6882-1-3 11513758 PMC37539

[B32] Guidotti M Mauri M Barrila C Guidotti F Belloni C Frovatriptan vs. transdermal oestrogens or naproxen sodium for the prophylaxis of menstrual migraine J Headache Pain 2007 8 283 288 10.1007/s10194-007-0417-4 17955167 PMC3476156

[B33] North American Menopause Society Estrogen and progestogen use in postmenopausal women: 2010 position statement of The North American Menopause Society Menopause 2010 17 242 255 10.1097/gme.0b013e3181d0f6b9 20154637

[B34] Allais G Bussone G De Lorenzo C Castagnoli GI Zonca M Mana O Borgogno P Acuto G Benedetto C Naproxen sodium in short-term prophylaxis of pure menstrual migraine: pathophysiological and clinical considerations Neurol Sci 2007 Suppl 2 S225 S228 17508177 10.1007/s10072-007-0783-3

[B35] Ernst E Acupuncture research: where are the problems? Acupunct Med 1994 3 93 97

[B36] Lewith GT Machin D On the evaluation of the clinical effects of acupuncture Pain 1983 16 111 127 10.1016/0304-3959(83)90202-6 6348651

[B37] Mauskop A Alternative therapies in headache. Is there a role? Med Clin North Am 2001 85 1077 1084 10.1016/S0025-7125(05)70360-6 11480259

[B38] Huang MF Yu CD Placebo-control methods in clinical study of acupuncture and moxibustion abroad Zhongguo Zhen Jiu 2003 23 589 591

[B39] Alecrim-Andrade J Maciel-Junior JA Carne X Severino VG Correa-Filho HR Acupuncture in migraine prevention: a randomized sham controlled study with 6-months posttreatment follow-up Clin J Pain 2008 24 98 105 10.1097/AJP.0b013e3181590d66 18209514

[B40] Melchart D Streng A Hoppe A Brinkhaus B Witt C Wagenpfeil S Pfaffenrath V Hammes M Hummelsberger J Irnich D Weidenhammer W Willich SN Linde K Acupuncture in patients with tension-type headache: randomised controlled trial BMJ 2005 331 376 382 10.1136/bmj.38512.405440.8F 16055451 PMC1184247

[B41] Linde K Streng A Jurgens S Hoppe A Brinkhaus B Witt C Wagenpfeil S Pfaffenrath V Hammes MG Weidenhammer W Willich SN Melchart D Acupuncture for patients with migraine: a randomized controlled trial JAMA 2005 293 2118 2125 10.1001/jama.293.17.2118 15870415

