# Supplementary material for: Acupuncture as prophylaxis for menstrual-related migraine: study protocol for a multicenter randomized controlled trial
Source: Trials. 2013 Nov 6;14:374. doi: 10.1186/1745-6215-14-374 (PMC3830503; doi:10.1186/1745-6215-14-374)
Supplement: Additional file 1: Figure S1 — Study flow chart. [file 1745-6215-14-374-S1.doc]

Additional file 1

Figure S1: Study Flow Chart.

Patients Screened

Baseline Period

Patients excluded

Month: 0

Randomization

(184 patients estimated)

Data collection

In Baseline

Treatment group (n=92)

verum acupuncture and placebo medicine

Control Group (n=92)

sham acupuncture and medicine

(Naproxen Sustained Release Tablets)

Subgroup B Subgroup C Subgroup D

（n=31） （n=31） （n=30）

2 sessions per week,

3 months (menstrual cycles)

Month: 3

Telephone interviews

Month: 4

Follow up

for data collection

End of trial
