# Supplementary material for: Acupuncture as prophylaxis for menstrual-related migraine: study protocol for a multicenter randomized controlled trial
Source: Trials. 2013 Nov 6;14:374. doi: 10.1186/1745-6215-14-374 (PMC3830503; doi:10.1186/1745-6215-14-374)
Supplement: Additional file 2: Table S1 — Time to visit and data collection. [file 1745-6215-14-374-S2.doc]

Additional file 2

Table S1: Time to visit and data collection

| Measures | Baseline | Treatment Phase | | | Follow-up Phase |
| --- | --- | --- | --- | --- | --- |
|  |  | 1-Month | 2-Month | 3-Month | 4-Month |
| Informed consent | ╳ |  |  |  |  |
| Randomization | ╳ |  |  |  |  |
| Medical history | ╳ |  |  |  |  |
| Headache diary | ╳ | ╳ | ╳ | ╳ | ╳ |
| VAS | ╳ | ╳ | ╳ | ╳ | ╳ |
| Headache intensity grade | ╳ | ╳ | ╳ | ╳ | ╳ |
| Intake of acute-medication | ╳ | ╳ | ╳ | ╳ | ╳ |
| Adverse events |  | ╳ | ╳ | ╳ | ╳ |
| Blinding Test |  |  | ╳ | ╳ |  |
| Reasons of drop-outs or withdrawals |  | ╳ | ╳ | ╳ | ╳ |
| Patient’s compliance |  | ╳ | ╳ | ╳ | ╳ |

Note: VAS = visual analogue scale (0–10); Headache intensity grade (a four-point scale): 0 = no pain; 1 = mild pain; 2 = moderate pain; 3 = severe pain.
