# Supplementary material for: Acupuncture as prophylaxis for menstrual-related migraine: study protocol for a multicenter randomized controlled trial
Source: Trials. 2013 Nov 6;14:374. doi: 10.1186/1745-6215-14-374 (PMC3830503; doi:10.1186/1745-6215-14-374)
Supplement: Additional file 3: Table S2 — Fifteen points without effects on headache or menstruation. [file 1745-6215-14-374-S3.doc]

Additional file 3

Table S2: 15 points without effects on headache or menstruation

| Arm LU5 LI12 LI14 LI15 HT4 PC2 PC3 SJ8 |
| --- |
| Leg ST32 ST37 KI9 GB32 GB33 GB35 LR7 |
