# Supplementary material for: Acupuncture as prophylaxis for menstrual-related migraine: study protocol for a multicenter randomized controlled trial
Source: Trials. 2013 Nov 6;14:374. doi: 10.1186/1745-6215-14-374 (PMC3830503; doi:10.1186/1745-6215-14-374)
Supplement: Additional file 4: Table S3 — The sham points in the control group. [file 1745-6215-14-374-S4.doc]

Additional file 4

Table S3: The sham points in the control group

| Subgroup | Code | Arm | | Leg | | |
| --- | --- | --- | --- | --- | --- | --- |
| 1 | B | LU5 | HT4 | KI9 | GB33 | GB35 |
| 2 | C | LI15 | PC3 | GB35 | LR7 | ST32 |
| 3 | D | LI12 | LI14 | GB32 | ST37 | KI9 |
